# Troubled sleep: Night waking, breastfeeding and parent–offspring conflict

**DOI:** 10.1093/emph/eou005

**Published:** 2014-04-08

**Authors:** David Haig

**Affiliations:** Department of Organismic and Evolutionary Biology, Harvard University, 26 Oxford Street, Cambridge, MA 02138, USA

**Keywords:** lactational amenorrhea, interbirth intervals, night waking, breastfeeding, co-sleeping, evolutionary pediatrics

## Abstract

Disrupted sleep is probably the most common complaint of parents with a new baby. Night waking increases in the second half of the first year of infant life and is more pronounced for breastfed infants. Sleep-related phenotypes of infants with Prader-Willi and Angelman syndromes suggest that imprinted genes of paternal origin promote greater wakefulness whereas imprinted genes of maternal origin favor more consolidated sleep. All these observations are consistent with a hypothesis that waking at night to suckle is an adaptation of infants to extend their mothers’ lactational amenorrhea, thus delaying the birth of a younger sib and enhancing infant survival.

## INTRODUCTION

Parents with young children often complain of fragmented sleep. Pediatricians advise parents how babies can be trained to sleep through the night in their own crib [1] while anthropologists advocate co-sleeping and voice concern about ‘caring for human infants in ways that are not congruent with their evolutionary biology’ [2]. Fostering independence is opposed to strengthening attachment [3, 4]. Some view the disruption of parental sleep as a problem to be solved [5], whereas others view frequent night suckling in a shared bed as part of our evolutionary heritage with which we tamper at our peril [6, 7]. Arguments about how to care for infants have moral overtones with subliminal messages of good and bad mothers [8, 9], and of selfish parents putting their desire for a good night’s sleep above the needs of their infants.

Depictions of mothers with infants arouse deep feelings and evoke potent myths. Once there was a time of intimate physical contact and tight emotional bonding between mothers and infants, each secure in the other’s love, but paradise was lost through the temptations of modernity. Yet paradise can be regained if we return to ‘natural’ and ‘instinctive’ modes of parenting. This tale of Fall and Redemption is often cloaked in an appeal to our evolved nature. Myths contain truths. Mothers have evolved to care for and love their infants, but evolutionary theory distinguishes between health and fitness and predicts divergence of genetic interests between parents and offspring [10]. This article argues that the sleep of infants and complaints of parents can be partially illumined by attention to this divergence. The serpent was always in the garden.

Blurton Jones and da Costa proposed that night waking to suckle is an adaptation of infants to suppress ovarian function in their mothers, thereby delaying the conception of a younger sib with whom an infant must compete for parental care and attention [11]. Others have noted that suckling confers contraceptive, as well as nutritive, benefits [12, 13], but these authors’ distinctive contribution was to recognize that the optimal interbirth interval (IBI) for parents was shorter than the optimal IBI for offspring. No implication was intended that contraception was a conscious motivation of infants, but simply that infants who woke their mothers left more descendants. Neither was the resumption of ovulation implied to be a conscious maternal strategy to trade a decrement in probability of survival for an extra child, simply that more total offspring survived if IBIs were shorter than were best for the survival of individual infants.

Something is optimal if it maximizes the value of a ‘desired’ quantity. In evolutionary biology, this quantity is fitness but, in medicine, it is health. Evolutionary medicine must attend to both concepts of optimality but maintain a clear distinction between them. When we attempt to identify adaptive functions of evolved systems, we need to understand how an adaptation has contributed to fitness, sometimes in ways contrary to health. But when we consider the efficacy of medical interventions, we need to understand how actions contribute to health and human autonomy, regardless of consequences for fitness.

Maximization of fitness need not maximize well-being. Two quotations illustrate this distinction with respect to ‘optimal’ IBIs; the first from a monograph on Aché hunter–gatherers of Paraguay [14] and the second from a report of the World Fertility Survey [15].

Despite the fact that offspring survival is higher at intermediate fertility rates, the extra offspring produced by achieving short IBIs more than compensate for the increased rate of loss of those offspring.

For what it is worth, we note than any family trying to achieve maximal numbers of surviving children at any cost would, in the light of these results, continue to bear children at the most rapid rate possible. The dramatic excess mortality is not enough to negate the extra births. However, it is hard to recommend a pattern with such disastrous human consequences.

This trade-off between the number of surviving offspring and survival of individual offspring means that IBIs that maximized parental fitness were suboptimal for offspring fitness and vice versa.

Human mothers wean their infants at younger ages, and return to fertility sooner, than do our closest relatives. Thus, comparatively short IBIs are a derived feature of our life history that enabled us to produce offspring more rapidly than other great apes [16]. The offspring number/survival trade-off probably shifted in favor of shorter IBIs because of inputs of allomaternal care that reduced costs to mothers while enhancing child survival [17, 18], but such inputs did not alter the fundamental logic that costs and benefits were differentially weighted by genes in mothers and infants. The next section outlines theories of parent–offspring conflict and of conflict between genes of maternal and paternal origin within offspring genomes.

## INTERGENERATIONAL AND INTRAGENOMIC CONFLICT

As any parent will affirm, the more children one has the less one can provide for each in purely material terms, but parents are often reluctant to concede that similar trade-offs exist with respect to less material investments of time, care and attention. But an onlooker can testify that a mother encumbered with a babe in arms is less able to grab a toddler at heel as the older child stumbles into danger.

Robert Trivers formalized intergenerational trade-offs in his theory of parent–offspring conflict. He defined parental investment as an opportunity cost, ‘anything done by the parent for the offspring that increases the offspring’s chance of surviving while decreasing the parent’s ability to invest in other offspring’ [10]. Defined in this manner, parent–offspring conflict can be seen as a manifestation of sibling rivalry (something parents readily acknowledge) mediated through preemption of parental investment. In Trivers’ simple model, an increment of parental investment was of benefit (*B*) to the current child but of cost (*C*) to the parent’s other offspring. A gene in the parent had an even chance of being present in each offspring. Therefore, such a gene profited from the investment if *B*> *C*. By contrast, a gene in the child received the benefit with certainty but had only a chance, measured by the relatedness *r* of sibs, of being present in other offspring who suffered the opportunity cost. Therefore, a gene in the child would profit if *B*> *rC*. The difference in exchange rates of costs and benefits for genes in parents and offspring implied that genes in offspring would benefit from investment in their child despite a decrease in parental fitness for benefit–cost ratios in the range

r<B/C<1.

For benefit–cost ratios outside this range, there is a harmony of interests between parent and offspring. Both agree that the offspring should receive the benefit if *B*> *C* and both agree that the offspring should forgo the benefit if *B*< *rC*.

The value of *r* in Trivers’ model averaged distinct probabilities that alleles of maternal and paternal origin would be present in the offspring experiencing the opportunity cost, *r*= (*r*_m_+ *r*_p_)/2. In the context of the costs and benefits of maternal investment, *r*_m_= 0.5 but *r*_p_< 0.5 because mothers sometimes have offspring by multiple fathers [19]. Therefore, genes of paternal and maternal origin in offspring will ‘disagree’ about whether to impose a cost on the mother for a benefit to the offspring whenever benefit–cost ratios fall in the range

$$r_{\text{p}}<B/C<r_{\text{m}}.$$

The use of the average coefficient of relatedness *r* is justified if a gene’s effects are independent of its parental origin, because the best a gene can do when it lacks information about whether it occupies a maternal or paternal role is to adopt the compromise that does best, on average, across the two roles. But the use of *r*_m_ and *r*_p_ is appropriate when a gene possesses information about its parental origin, as occurs at imprinted loci. In this case, a gene’s best strategy is to act differently in the two roles [20].

The above analysis illustrates fundamental similarities between hypotheses of parent–offspring conflict and parental conflict within offspring genomes. The trade-off measured by *B*/*C* is the same, but the hypotheses differ by whether genes of offspring possess information about their sex of origin. Conflict between paternal and maternal alleles at imprinted loci is evidence that the trade-off has been evolutionarily significant and therefore supports the existence of parent–offspring conflict at unimprinted loci.

Most biologists’ implicit model of physiological systems is that parts are coordinated to achieve optimal function and that pathology results from malfunction, either because a part is broken or because a system is asked to perform under conditions outside of its evolutionary specifications (environmental mismatch). This model is inadequate for parent–offspring relations considered as a physiological system because ‘optimal’ varies for different genes in the system. Parts may work at cross-purposes. Function for one may be malfunction for another, and the system, as a whole, need not evolve toward more efficient outcomes [21]. In the next section, I present evidence that infant sleep and suckling behavior have been foci of evolutionary conflict both between genes expressed in mothers and genes expressed in infants and between genes of maternal and paternal origin within infant genomes.

## SUCKLING AND SLEEP

Short delays until the birth of a younger sib are associated with increased mortality of infants and toddlers, especially in environments of resource scarcity and rampant infectious disease. Costs are greatest for conception during the first year of postnatal life, with birth of a sib 9 months later [22–25]. Benefits of delay can be substantial: second-year mortality in rural Senegal was 16% with a birth in that year but 4% otherwise [26]. These selective forces are probably sufficiently strong to have engendered significant evolutionary responses since the adoption of agriculture.

The duration of postpartum amenorrhea is a major determinant of IBI in natural fertility populations [27] with more frequent and more intense nursing, especially at night, associated with prolonged infertility [28–30]. Natural selection will have preserved suckling and sleeping behaviors of infants that suppress ovarian function in mothers because infants have benefited from delay of the next birth. This proposed adaptation is independent of whether ‘suckling intensity’ or ‘metabolic load’ is the proximate cause of anovulation because lactation is energetically costly and suckling is one of the most direct ways an infant can increase its mother’s metabolic load [31, 32]. Maternal fatigue can be seen as an integral part of an infant’s strategy to extend the IBI.

Few breastfeeding mothers in our evolutionary past would have ovulated within the first few months postpartum, regardless of the precise pattern of infant suckling, but intergenerational conflict would have escalated at child ages at which mothers began to return to fertility and then have diminished as the benefit-to-cost ratio of maternal infertility declined. Maximal night waking can be conjectured to overlap with the greatest benefits of contraceptive suckling. Consistent with this expectation, infant sleep becomes more fragmented after 6 months and then gradually consolidates [33–37].

Weaned or bottle-fed infants wake less often at night than breast-fed infants [38–44] and weaning is reported, at least anecdotally, to reduce night waking and alleviate complaints of parents [33]. Sleep problems in Swedish infants were associated with breastfeeding at night and refusal of solid foods [37]. An association of breastfeeding with night waking is also observed among Thai infants who habitually sleep with their parents [45]. Therefore, the association is not simply a cultural artifact of western sleeping arrangements. Breastfeeding has many virtues but, for many mothers, a good night’s sleep is not counted among them.

If the function of night waking is to prolong lactational amenorrhea, and uninterrupted sleep has countervailing benefits, then waking would be maladaptive for infants whose mothers do not respond by nursing. The earlier onset of sleeping through the night in the absence of breastfeeding could thus be interpreted as the facultative quiescence of an ineffective function. Such an interpretation would imply that modern infants distinguish bottle-feeding from suckling. The less-fragmented sleep of bottle-fed infants is often attributed to cow’s milk and formulae being more soporific than breast milk because less easily digested [46–48]. However, breastfed infants who are not nursed at night sleep longer than breastfed infants who are nursed at night even though both consume human milk [5, 49]. Waking, it would seem, is reinforced by breastfeeding and extinguished by the cessation of night suckling.

If unimprinted genes of infants have been selected to extend IBIs beyond the maternal optimum through waking and suckling at night, then imprinted genes of maternal and paternal origin within offspring genomes would be predicted to have antagonistic effects on these same phenotypes. Prader-Willi (PWS) and Angelman (AS) syndromes are caused by deletion of a cluster of imprinted genes at chromosome 15q13 but differ in the parental origin of the deletion. The paternally inherited cluster is deleted in PWS but the maternally inherited cluster in AS [50]. Infants with PWS have a feeble suck, weak cry and sleep a lot [51], whereas infants with AS wake frequently at night [52]. These phenotypes suggest that imprinted genes of paternal and maternal origin have contrasting effects on sleep in infants without deletions, with genes of paternal origin promoting suckling and waking. Small-scale behavioral interventions in which parents were instructed not to respond to night waking by children with AS have resulted in dramatic improvements in sleep quality [53, 54].

These effects of imprinted genes are consistent with a hypothesis that genes of paternal origin in infants have been selected to favor longer IBIs than genes of maternal origin. From this perspective, no unbroken interval of sleep is optimal for all genes of infant genomes: instead, the evolution of infant sleep has been buffeted by selection for competing optima. The distinctive properties of infant sleep are usually interpreted as stages in the maturation of neural circuitry and synaptic connections. Effects of imprinted genes suggest that sleep maturation may not be a purely harmonious process.

Maternally expressed genes, paternally expressed genes and unimprinted genes have been selected for different degrees of wakefulness and this ‘disagreement’ may be reflected in a certain disorder in processes of falling and staying asleep that should resolve as intragenomic conflict lessens with age and the child ‘learns’ to sleep through the night. The architecture of infant sleep can be likened to a ramshackle structure put together by a committee from contradictory plans. Effects of imprinted genes also indirectly support the hypothesis that unimprinted genes in offspring promote longer IBIs than are optimal for mothers.

Human breast milk is a complex cocktail of nutrients and bioactive molecules [55]. Because milk is a maternal product, its composition and quantity are expected to have evolved to maximize maternal inclusive fitness subject to nutritional constraints of mothers. If infants evolved to wake more often than was evolutionarily optimal for mothers then mothers would have evolved counteradaptations some of which might be expressed in properties of milk.

A glass of warm milk before bed is commonly believed to facilitate falling asleep. Milk is highly nutritious but slowly digested. Caseins clot in the stomach, delaying gastric emptying, with a prolonged release of amino acids and peptides in the intestine [56, 57]. These studies involved adult volunteers ingesting bovine caseins that form a very thick curd. Human caseins form a much finer curd and exit the infant stomach more rapidly than bovine caseins [58, 59]. Unfortunately little is known about the time course of intestinal digestion of human milk proteins.

Among the peptides released from human caseins are β-casomorphins that bind to opioid receptors [60] and have been reported to enter infant blood and cross the blood–brain barrier [61]. Their biological functions in human infants are unknown, but experiments with rat pups found β-casomorphins increased quiet sleep [62] and reduced gut motility, with an associated increase in gastrointestinal transit times [63].

Breast milk contains hormones that regulate appetite and metabolism and that have been postulated to have long-term benefits for infants [64]. These hormones could be considered a form of maternal metabolic guidance for the infant. Mother’s milk is often considered an unimprovable infant food but this should not be an unquestioned axiom. Maternal and filial inclusive fitness broadly overlap but are not identical.

## THE MILK OF HUMAN KINDNESS

‘Evolutionary medicine takes the view that many contemporary social, psychological, and physical ills are related to incompatibility between the lifestyles and environments in which humans currently live and the conditions under which human biology evolved.’ [65]

The epigraph illustrates a strand of thought within evolutionary medicine that ascribes current woes to disparities between modern life and ancestral environments. Some of these discontents, it is suggested, would be remedied if our lives were more in harmony with our evolved nature. An anthropological school of evolutionary pediatrics emphasizes mismatches between optimal conditions for child development and ‘contemporary Euro-American infant care practices’. Its practitioners challenge ‘clinical wisdom regarding “normal” infant sleep’ and ‘the supremacy of pediatric sleep medicine in defining what are appropriate sleep environments and behaviors for healthy human infants’ [2]. The school has been productive of testable hypotheses, empirical research on mother–infant interactions and acrimonious exchanges about safe sleeping environments [12, 66, 67].

Some environmental mismatches enhance well-being. Childhood evolved under conditions in which malnutrition, infectious disease and accidents were major causes of mortality, but the fortunate infants of affluent countries live in a novel environment in which starvation and deaths from pathogens are rare, in which life expectancy approaches or exceeds 80 years, in which pediatric advice against co-sleeping aims to reduce risks of tragic but rare events, and in which evolutionary pediatrics concerns itself with subtle effects of alternative infant care on psychological health. Modern pediatrics and public health have achieved historically low rates of infant mortality and should be given the credit they deserve.

The school of evolutionary pediatrics systematically neglects considerations of intergenerational conflict. A quotation gives the flavor: ‘Infant needs, and parental responses to those needs, constitute a dynamic, co-evolving interdependent system shaped and designed by natural selection to maximize the chances of infant survival and, hence, parental reproductive success’ [68]. Infant needs and parental responses are indeed dynamic and interdependent but maternal fitness is not maximized by maximizing offspring fitness because infant survival trades-off against number of surviving offspring mediated via effects on IBIs. Evolutionary theory and demographic data converge on the conclusion that the inclusive fitness of infants is maximized by IBIs longer than those that maximize the inclusive fitness of mothers.

What implications do theories of parent–offspring conflict have for an expanded discipline of evolutionary pediatrics? The standard justification of basic research in the health sciences is as valid (or as self-serving) for evolutionary biology as it is for molecular and cellular biology: a better understanding of the processes that have shaped physiology and behavior will eventually facilitate more effective interventions. However, assumptions about what is ‘natural’ already influence advice to parents on how best to care for infants and are invoked by empirical scientists and clinicians in concepts of ‘normal’ function. Therefore, the immediate relevance of these theories is to identify assumptions that misrepresent evolutionary processes and may cause unnecessary anxiety to parents.

Problems of infant sleep are major parental concerns not only in Euro-American societies [69]. This article revives a hypothesis of Blurton Jones and da Costa that night waking is, in part, an adaptation of infants to extend IBIs. In the developed world, many of the health advantages of prolonged IBIs have diminished and more reliable forms of contraception have replaced lactational amenorrhea. Therefore, the selective forces responsible for these behaviors have been attenuated but the behaviors remain part of our biological heritage. One should question whether modern sleep practices have had unintended consequences for child health but it would be irresponsible to recommend changes to these practices, solely on the basis of mismatch, without epidemiological evidence of harm. Mismatch is a medical problem only if it causes pathology.

I am not competent to suggest policy on complex public health issues of infant sleep and feeding, but can offer two bits of evolutionary counsel to parents. First, some degree of tension between needs of parents and infants is what one might expect from evolutionary theory. Second, evolutionary logic suggests child development should be robust and adaptable with respect to factors that were variable in the evolutionary past. Moreover, natural selection will have favored a degree of adaptability of both parents and offspring to novel environments because the past never repeats precisely. The comforting news is that child well-being is unlikely to be irrevocably compromised by minor variations in parental care.

Identification of the ‘environment of evolutionary adaptedness’ with the optimal environment for well-being conflates questions of fitness and health. We did not evolve to be happy or healthy, but to be fit, and to be happy, miserable, kind, callous, generous and vindictive as proximate means to the end of fitness. We can aspire to the positive among our repertoire of adaptations while abhoring the negative, and we can aspire to collective health and well-being. There is no lost Eden of perfect harmony between mother and child. What was best for one was not always best for the other. They never were one body and one flesh. Genetic conflicts within the family are part of our biological heritage, as are love and care for our children.
